# A Homochiral Poly(2‐oxazoline)‐based Membrane for Efficient Enantioselective Separation

**DOI:** 10.1002/anie.202212139

**Published:** 2023-01-18

**Authors:** Fanmengjing Wang, David Pizzi, Yizhihao Lu, Kaiqiang He, Kristofer J. Thurecht, Matthew R. Hill, Philip J. Marriott, Mark M. Banaszak Holl, Kristian Kempe, Huanting Wang

**Affiliations:** ^1^ Department of Chemical and Biological Engineering Monash University 3800 Clayton Victoria Australia; ^2^ Drug Delivery Disposition and Dynamics Monash Institute of Pharmaceutical Sciences Monash University 3052 Parkville VIC Australia; ^3^ CAS Key Laboratory of Bio-Inspired Materials and Interfacial Science Technical Institute of Physics and Chemistry Chinese Academy of Sciences 100190 Beijing P. R. China; ^4^ Centre for Advanced Imaging (CAI) and Australian Institute for Bioengineering and Nanotechnology ARC Training Centre for Innovation in Biomedical Imaging Technology The University of Queensland 4072 St. Lucia QLD Australia; ^5^ School of Chemistry Monash University 3800 Clayton Victoria Australia; ^6^ Materials Science and Engineering Monash University 3800 Clayton Victoria Australia

**Keywords:** Chiral Resolution, Chirality, Homochiral Polymers, Membranes, Poly(2-Oxazoline)s

## Abstract

Chiral separation membranes have shown great potential for the efficient separation of racemic mixtures into enantiopure components for many applications, such as in the food and pharmaceutical industries; however, scalable fabrication of membranes with both high enantioselectivity and flux remains a challenge. Herein, enantiopure *S*‐poly(2,4‐dimethyl‐2‐oxazoline) (*S*‐PdMeOx) macromonomers were synthesized and used to prepare a new type of enantioselective membrane consisting of a chiral *S*‐PdMeOx network scaffolded by graphene oxide (GO) nanosheets. The *S*‐PdMeOx‐based membrane showed a near‐quantitative enantiomeric excess (*ee*) (98.3±1.7 %) of *S*‐(−)‐limonene over *R*‐(+)‐limonene and a flux of 0.32 mmol m^−2^ h^−1^. This work demonstrates the potential of homochiral poly(2,4‐disubstituted‐2‐oxazoline)s in chiral discrimination and provides a new route to the development of highly efficient enantioselective membranes using synthetic homochiral polymer networks.

## Graphical abstract

*S*‐Poly(2,4‐dimethyl‐2‐oxazoline)‐based membranes (*S*‐PdMeOx/GO) are prepared via a simple solution‐based method on porous substrates for chiral separation. The *S*‐PdMeOx‐based network within the graphene oxide scaffold induced homochirality in the membrane. The resulting *S*‐PdMeOx‐based membrane displays excellent enantioselectivity in separating racemic limonene mixtures.

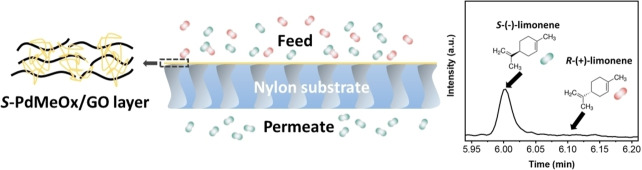

Enantiomers of a chiral molecule show distinct pharmaceutical or biological activities toward inherently chiral organisms; therefore, it is of great significance to obtain enantiopure molecules for pharmaceutical manufacturing, food and other applications.^[1]^ Membrane‐assisted enantioselective separation is an attractive method of harvesting enantiomers in a continuous mode and can be easily scaled up.^[2]^ Polymers with homochirality are a dominant class of materials for chiral separation membranes due to their good processability. Among the enantiopure polymeric materials studied to date, natural polysaccharides, especially cellulose, chitosan, and their derivatives, are popular choices for constructing chiral separation membranes because of their highly regular structures and unique helical conformations.^[3]^ A pair of enantiomers pass through the membrane at different rates due to the difference in affinities between the enantiomer and the chiral moieties within the membrane, which makes the membrane enantioselective.^[2b]^ Polysaccharides derive their chirality from their carbon centers in the main chain; however, racemic molecules tend to encounter flexible side chains during penetration, which makes it a challenge for polysaccharide‐based membranes to achieve high enantioselectivity for separating racemic compounds.^[4]^ Functionalizing polymeric membranes with additional chiral additives, such as β‐cyclodextrins (β‐CD) and amino acids, is an alternative method that has been widely reported; however, it is difficult to control the efficiency of post‐modification.^[5]^ Moreover, achieving high enantioselectivity while maintaining a satisfactory flux of enantiomers remains a challenge for polymeric membranes; therefore, designing new homochiral networks for efficient membrane‐based chiral separation is highly desirable.

Poly(2‐oxazoline)s (POx) are a class of polymeric materials that are renowned for their ease of synthesis and structural and chemical diversity.^[6]^ Importantly, the introduction of functional groups in the 2‐position of the 2‐oxazoline monomer, which will form side chains in the resulting polymer, is considered the major strategy to tune the polymer's physicochemical properties.^[7]^ Generally, short aliphatic side chains such as methyl or ethyl groups give access to water‐soluble POx, while long aliphatic or aromatic side chains render POx hydrophobic.^[8]^ Similarly, the use of functional initiators and termination agents in the living cationic ring‐opening polymerization (CROP) of 2‐oxazolines allows the introduction of further functionalities at the α,ω‐end of POx.^[9]^ This has been exploited for the synthesis of POx macromonomers (MMs) through the attachment of polymerizable end groups. In recent years, substantial effort has been made to build more complex architectures with broader functionalities using POx.^[10]^ Thus, POx represents a highly functional polymer platform with wide application potential.^[11]^ However, modifications of the 4‐ or 5‐position of the 2‐oxazoline have only received very limited attention, despite the enormous potential to introduce a chiral center in the POx backbone when polymerizing 4‐/5‐substituted‐2‐oxazolines. To date, attention has been mainly focused on hydrophobic, optically active, poly(2,4‐substituted‐2‐oxazoline)s, particularly with regard to their synthesis and potential structures and use as core components of POx‐based micelles.^[12]^ The formation of a secondary structure in hydrophobic chiral POx was confirmed in both solution and the solid‐state using techniques including differential scanning calorimetry (DSC), X‐ray diffraction (XRD), and circular dichroism (CD).^[12]^ More recently, the effect of 4‐substitution on the more hydrophobic POx as a core block in micelle formulation has been investigated to probe the chiral specific interactions of polymer and drugs in the context of drug loading.^[13]^ In contrast, there is only one known report on water‐soluble chiral poly(2,4‐substituted‐2‐oxazoline)s, which confirmed via CD spectroscopy that poly(2,4‐dimethyl‐2‐oxazoline) (PdMeOx) formed temperature‐sensitive helices in aqueous solutions.^[14]^

Herein, we report a new type of enantioselective polymeric membrane derived from an *S*‐poly(2,4‐dimethyl‐2‐oxazoline) (*S*‐PdMeOx)‐based crosslinked network that contains a large number of pendant, functionalized short polymer chains. Water‐soluble *S*‐PdMeOx macromonomers (*S*‐PdMeOx_5_A) were synthesized via a living CROP termination strategy (Scheme 1a) and subsequently polymerized via radical polymerization in the presence of *N,N*′‐methylenebisacrylamide (MBA) as a crosslinker (Scheme 1b). Due to the high water solubility of *S*‐PdMeOx macromonomers, graphene oxide (GO) nanosheets were used as a scaffold to facilitate membrane formation (Figure 1a). Within the polymeric membrane, the homochiral *S*‐PdMeOx‐based network provided densely distributed chiral sites by wrapping up and filling in between the GO layers. Enantioselective transport of limonene enantiomers through *S*‐PdMeOx/GO composite nanochannels was achieved. *S*‐(−)‐limonene molecules were found in excess in the permeate with a flux of 0.32 mmol m^−2^ h^−1^ and an enantiomeric excess (*ee*) value of 98.3±1.7 %.

**Scheme 1:**
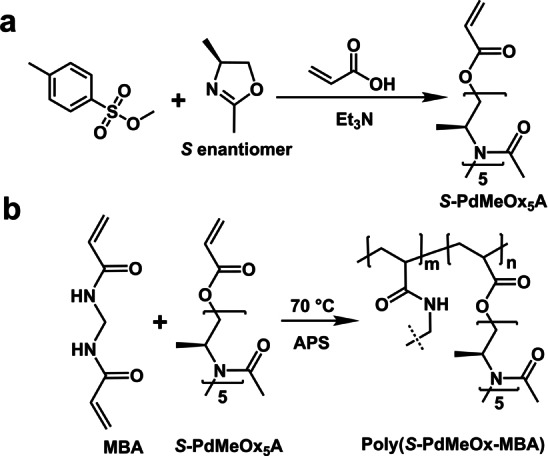
a) Synthesis of *S*‐poly(2,4‐dimethyl 2‐oxazoline) (*S*‐PdMeOx) macromonomer. b) Synthesis of crosslinked (*S*‐PdMeOx‐MBA) network.

**Figure 1:**
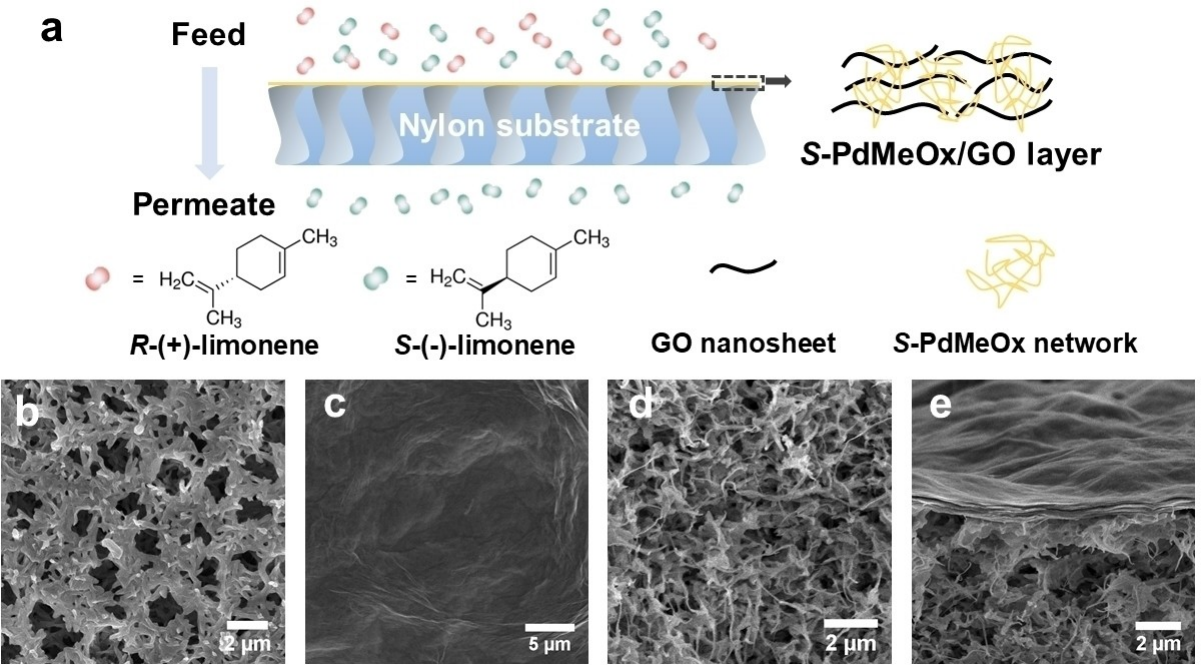
a) Illustration of the structure of the *S*‐PdMeOx/GO membrane and the enantioselective transport of limonene enantiomers through the membrane. b)–e) SEM images of surface morphologies and cross‐sections of nylon substrate (b) and (d) and *S*‐PdMeOx/GO membrane (c) and (e), respectively.

2,4‐dimethyl‐2‐oxazoline (dMeOx) monomers (both enantiopure and racemic) were prepared and purified as described in the literature.^[14]^ These monomers were then polymerized via CROP into acrylate‐terminated MMs (*S*‐PdMeOx_5_A and *RS*‐PdMeOx_5_A) (Scheme 1a and Supporting Information Scheme S1). Successful synthesis and end group fidelity of *S*‐PdMeOx_5_A and the *RS*‐PdMeOx_5_A control were confirmed by ^1^H nuclear magnetic resonance (^1^H NMR) analysis (Supporting Information Figure S1 and S2). CD spectroscopy was performed to confirm the homochirality of the *S*‐PdMeOx_5_A MMs. Indeed, a notable Cotton effect was observed at an adsorption peak of 230 nm in the CD spectrum of *S*‐PdMeOx_5_A, indicating the presence of a chiral structure. As expected, the spectrum of the control system *RS*‐PdMeOx_5_A (Figure 2a) produced no signal, confirming its racemic nature. Size exclusion chromatography (SEC) analysis demonstrated products with low molecular weight dispersity that showed good overlap between the two prepared systems (Figure 2b), further suggesting that the targeted length was achieved and that the MMs were otherwise comparable, minimizing any nonchiral effects (Supporting Information Table S1).

**Figure 2:**
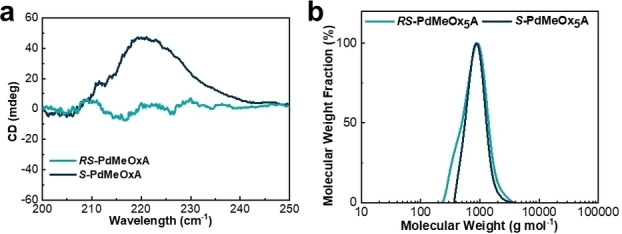
Characterization of the *S*‐PdMeOx_5_A and *RS*‐PdMeOx_5_A macromonomers. a) CD spectra; and b) SEC traces.

The *S*‐poly(2,4‐dimethyl‐2‐oxazoline)/graphene oxide (*S*‐PdMeOx/GO) membrane (Figure 1a and Supporting Information Figure S3) was prepared via a simple two‐step solution‐based casting method. A porous nylon support was first coated with a precursor solution consisting of homochiral *S*‐PdMeOx_5_A macromonomers, crosslinker (MBA), initiator ammonium persulfate (APS), and GO nanosheets via vacuum filtration, followed by free‐radical polymerization of the macromonomer at 70 °C. The cast substrate was then coated with a polymer‐GO layer again by spin coating of the precursor solution and subsequent polymerization at 70 °C. During the vacuum filtration and spin coating processes, the GO laminate served as a scaffold for membrane formation, and the monomer solution was confined within the interlayers of nanosheets. Polymerization occurred between the laminate layers, and the homochiral network poly(*S*‐PdMeOx‐MBA) that entwined the GO nanosheets was formed (Scheme 1b), leading to a defect‐free composite membrane. As shown in the scanning electron microscope (SEM) images of the surface morphologies and cross‐sections of the pristine nylon (Figure 1b and d) and *S*‐PdMeOx/GO membrane (Figure 1c and e), defect‐free and uniform polymer‐GO active layers were successfully coated onto the porous substrate. The thickness of the composite layers was estimated using the cross‐section image of the membrane (Figure 1e), which is approximately 0.59 μm. From the XRD patterns of pure GO laminates and the composite membrane in the dry state (Figure 3a), it was found that the layer spacing of GO laminates increased from 0.75 to 0.94 nm after the successful incorporation of the homochiral polymer network. The layer spacing of the membrane stabilized to 0.97 nm in the wet state, creating confined nanochannels, which facilitate a strong interaction between enantiomers and chiral recognition sites. The chemical composition of the polymeric membrane was confirmed using Fourier transform infrared spectroscopy (FT‐IR) analysis (Supporting Information Figure S4). Characteristic peaks of both graphene oxide and the polymer network were identified in the spectrum of the *S*‐PdMeOx/GO membrane. No additional peaks were identified in the spectrum of the membrane other than the peaks of the polymer and GO nanosheets, suggesting that no chemical bonds were formed between graphene oxide and the crosslinked network. Moreover, thermogravimetric analysis (TGA) was used to determine the mass ratio of GO and the polymer network in the membrane. As shown in Figure 3b, the polymer networks were thermally stable until approximately 250 °C and started decomposing at temperatures greater than 350 °C. A mass loss of GO occurred between 100 °C and 250 °C, and there was 44 % residual graphene up to 700 °C. Considering the mass change and decomposition of the nylon support at around 400 °C and 550 °C, the mass ratio of GO and the polymer network in the *S*‐PdMeOx/GO membrane was calculated to be 6 : 5 by comparing the relative mass loss at around 150 °C (19 %) together with the 5 % graphene residue at 700 °C and the relative mass loss at around 300 °C (18.5 %).

**Figure 3:**
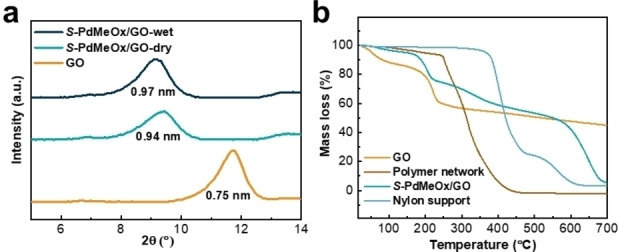
Characterization of the *S*‐PdMeOx/GO membrane. a) XRD patterns in dry and wet state. b) TGA curves of GO, *S*‐PdMeOx network, nylon support, and *S*‐PdMeOx/GO membrane.

To probe the performance of the new *S*‐PdMeOx/GO membrane, limonene enantiomers were chosen for separation. *S*‐(−)‐Limonene is less abundant in nature than *R*‐(+)‐limonene, which is found in citrus. Due to the pine‐like odor of *S*‐(−)‐limonene, it has a high demand in the food flavoring and fragrance industries. In addition, it has found application in industrial cleaning and has recently been described as an intermediate in the production of unnatural terpenes in engineered biosynthesis,^[15]^ making it a highly relevant model pair of compounds to separate. The enantioselective resolution capacity of the *S*‐PdMeOx‐based membrane was investigated through concentration‐driven separation tests using a 0.01 mol L^−1^ mixture of *R‐* and *S*‐limonene in ethanol as the feed solution. GC analyses of the permeates were conducted to determine the enantioselectivity of the composite membrane. As shown in Figure 4a and b, the intensity of responses to *S*‐enantiomers detected in the permeates at 2 h and 4 h were much higher than that of the *R‐*enantiomers (Supporting Information Figure S5), demonstrating the high resolution (*ee=*98.3±1.7 %) of *S*‐limonene over *R*‐limonene. The highest flux of *S*‐limonene molecules was reached at the initial stage of separation (2 h) with 0.32 mmol m^−2^ h^−1^, and limited nonselective permeation of *R*‐enantiomers was detected during separation (Supporting Information Table S2). To confirm that the enantioselectivity of the membrane originates from the addition of the *S*‐PdMeOx polymer network, control experiments were performed with the nylon substrate, pure GO nanosheets (supported by nylon substrate), and the achiral *RS*‐PdMeOx/GO membrane. Importantly, GC results showed that both enantiomers of limonene permeated through each of the control samples at similar rates and hence were not separated (Supporting Information Figure S6). To further investigate the separation mechanism, adsorption of *R*/*S*‐limonene by *S*‐PdMeOx/GO active layers was measured, and the result showed that there was no selective adsorption (Supporting Information Figure S7), which indicated that this composite membrane followed the selective diffusion‐permeation mechanism.[^16^, ^17^]

**Figure 4:**
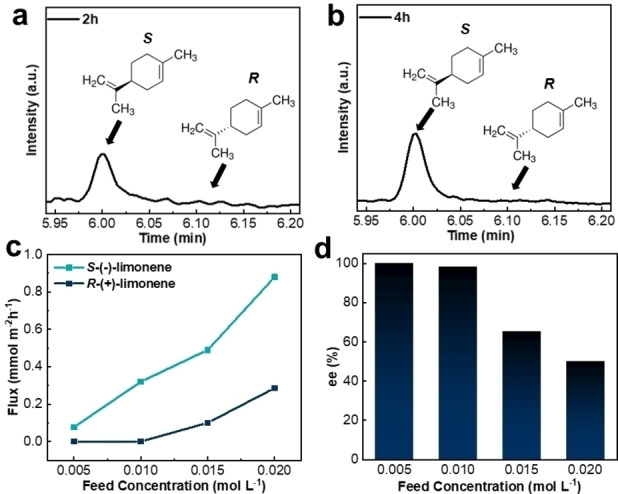
Enantioselective separation performance of the *S*‐PdMeOx/GO membrane. Gas chromatograms of resolved limonene enantiomers after separation at a) 2 h and b) 4 h (Conditions: 0.01 mol L^−1^ racemic *R*/*S*‐limonene in ethanol, room temperature. c) Flux of enantiomers of limonene through *S*‐PdMeOx/GO membrane (Conditions: 0.005 mol L^−1^, 0.01 mol L^−1^, 0.015 mol L^−1^, and 0.02 mol L^−1^ *R*/*S*‐limonene in ethanol, room temperature, 2 h separation. d) Chiral separation results of *S*‐PdMeOx/GO membrane (Conditions: 0.005 mol L^−1^, 0.01 mol L^−1^, 0.015 mol L^−1^, and 0.02 mol L^−1^ *R*/*S*‐limonene in ethanol, room temperature, 2 h separation).

To evaluate in more detail the effect of the feed solution concentration on the chiral separation performance of the *S*‐PdMeOx/GO membrane, *R*/*S*‐limonene/ethanol feed solutions with three different concentrations (0.005, 0.015 and 0.02 mol L^−1^) were examined. Figure 4c and d show the change in flux of *S*‐ and *R*‐enantiomers and *ee* value for the *S*‐PdMeOx/GO membrane as a function of feed solution concentration, respectively. The flux of *S*‐enantiomers was enhanced from 0.08 to 0.88 mmol m^−2^ h^−1^ with increasing feed concentrations from 0.005 to 0.02 mol L^−1^ (Supporting Information Table S2). This finding is consistent with the expectation that flux increases with increasing driving force, which is the concentration of feed solution in this work. However, the non‐selective diffusion of *R‐*enantiomers also increased at a higher concentration gradient; therefore, as shown in Figure 4d, high separation performance was achieved only under conditions of lower feed concentrations (0.005 and 0.01 M), and the enantioselectivity decreased to 65.3 % *ee* (0.015 M) and 50 % *ee* (0.02 M) with increasing feed concentration (Supporting Information Figure S8, Table S2). The results illustrated that a feed concentration of 0.01 mol L^−1^ was the optimum condition for achieving both high enantioselectivity and flux for the *S*‐PdMeOx/GO membrane.

To investigate the effect of the carrier solvent on the performance of the *S*‐PdMeOx/GO membrane, concentration‐driven separation experiments were carried out using hexane as the solvent of the feed solution at an optimized concentration of 0.01 mol L^−1^. In the presence of hexane, the *S*‐PdMeOx‐based network is most likely collapsed; therefore, it is worth exploring the performance of the *S*‐PdMeOx/GO membrane under this environment. Separation of the limonene enantiomers with hexane as the carrier solvent was achieved with *S*‐limonene preferentially permeating through the membrane. The GC results showed that the separation capacity was 54.6 % *ee*, and the flux of *S*‐limonene molecules with hexane as the solvent was 0.18 mmol m^−2^ h^−1^ (Supporting Information Figure S9). The separation capacity of the *S*‐PdMeOx/GO membrane was retained when using hexane as the solvent, even though due to the collapse of the polymer network, the performance decreased compared to using ethanol as the solvent.

Overall, the *S*‐PdMeOx/GO membrane simultaneously possesses outstanding enantioselectivity and flux compared with other enantioselective membranes (Supporting Information Table S3). An improvement in selectivity is seen for the separation of limonene racemates compared to the previously reported graphite phase carbon nitride‐based membrane.^[18]^ Among the current advanced polymeric, 2D material‐based, and nanoporous material‐based membranes for chiral separation, the *S*‐PdMeOx‐based membrane demonstrated superior enantioselectivity with comparable flux (Supporting Information Table S3).[^16^, ^17^, ^19^, ^20^] When compared to high‐performance polysaccharide membranes and post functionalized‐polymeric membranes, the *S*‐PdMeOx membrane exhibited the highest selectivity for chiral separation.[^3a^, ^3c^, ^5b^, ^21^] Due to the excellent separation performance and ease of fabrication of *S‐*PdMeOx/GO membranes, they are promising candidates for chiral separation applications.

In summary, we designed and prepared a novel *S*‐PdMeOx‐based enantioselective membrane for chiral resolution of racemic limonene mixtures, where the membrane displayed ultimate selectivity at room temperature. In the fabrication of the *S*‐PdMeOx/GO membrane, water‐soluble *S*‐PdMeOx_5_A macromonomers were readily introduced into GO matrixes, and enantiopure crosslinked networks were built with the addition of crosslinkers. Adsorption tests revealed that the *S*‐PdMeOx/GO membrane followed the selective diffusion‐permeation mechanism, and further experiments showed that the enantioselectivity of the membrane increased with decreasing feed concentration. In addition, optimized conditions were determined for this composite membrane to achieve high separation performance (98.3±1.7 % *ee*) with a feed concentration of 0.01 mol L^−1^ and ethanol as the carrier solvent. This work introduces water‐soluble, homochiral poly(2,4‐disubstituted‐2‐oxazoline)s as highly potent membrane components for next‐generation enantioselective membranes with high performance.

## Conflict of interest

The authors declare no conflict of interest.

## Supporting information

As a service to our authors and readers, this journal provides supporting information supplied by the authors. Such materials are peer reviewed and may be re‐organized for online delivery, but are not copy‐edited or typeset. Technical support issues arising from supporting information (other than missing files) should be addressed to the authors.

Supporting Information

## Data Availability

The data that support the findings of this study are available from the corresponding author upon reasonable request.
