# Supplementary material for: A Homochiral Poly(2‐oxazoline)‐based Membrane for Efficient Enantioselective Separation
Source: Angew Chem Int Ed Engl. 2023 Jan 18;62(8):e202212139. doi: 10.1002/anie.202212139 (PMC10107185; doi:10.1002/anie.202212139)
Supplement: Supplementary file 1 — Supporting Information [file ANIE-62-0-s001.pdf]

Angewandte Chemie
Eine Zeitschrift der Gesellschaft Deutscher Chemiker

Supporting Information

A Homochiral Poly(2-oxazoline)-based Membrane for Efficient Enantioselective Separation

F. Wang, D. Pizzi, Y. Lu, K. He, K. J. Thurecht, M. R. Hill, P. J. Marriott, M. M. Banaszak Holl, K. Kempe, H. Wang**

Table of Contents

Scheme S1. Synthesis of racemic poly(2,4-dimethyl 2-oxazoline) (PdMeOx) macromonomer. 5
Figure S1. 1H NMR spectra (400 MHz, $CDCl_3$) of S-PdMeOx$_5$A. 5
Figure S2. 1H NMR spectra (400 MHz, D_2O) of RS-PeMeOx$_5$A. 5
Figure S3. Digital photo of S-PdMeOx/GO membranes. 6
Figure S4. The Fourier transform infrared (FT-IR) spectra of GO laminates (blue), the S-PdMeOx polymer network (black), and S-PdMeOx/GO membrane (red). 6
Figure S5. Gas chromatograms of 0.01 mol L^{-1} R/S-limonene/ethanol standard solution. 7
Figure S6. Gas chromatograms of resolved limonene molecules after 2 h using a) pure nylon substrate, b) GO nanosheets (supported by nylon substrate), and c) PdMeOx/GO membrane under the condition of 0.01 mol L^{-1} R/S-limonene/ethanol as feed solution at room temperature. 7
Figure S7. Gas chromatograms of R/S-limonene adsorbed by the S-PdMeOx/GO active layer (24 h). 7
Figure S8. Gas chromatograms of resolved limonene enantiomers after 2 h using the S-PdMeOx/GO membrane under the conditions of (a) 0.005 mol L^{-1}, (b) 0.015 mol L^{-1}, and c) 0.02 mol L^{-1} R/S-limonene/ethanol as feed solution at room temperature. 7
Figure S9. Gas chromatograms of resolved limonene enantiomers after 4 h using the S-PdMeOx/GO membrane under the conditions of 0.01 mol L^{-1} R/S-limonene/hexane as the feed solution at room temperature. 8
Table S1. Macromonomer molar masses and dispersity as measured by SEC. 8
Table S2. Enantioselectivity and solute flux of the S-PdMeOx/GO membrane with permeation time. 8
Table S3. Comparison of the chiral separation performance of the S-PdMeOx/GO membrane with reported representative enantioselective membranes. 8

Experimental Procedures

1. Materials

2-Aminopropan-1-ol (97%, AA BLOCKS), (*R*)-2-aminopropan-1-ol (98%, AA BLOCKS), (*S*)-2-aminopropan-1-ol (98%, AA BLOCKS), propionitrile (>99%, Sigma–Aldrich), acrylic acid (>99%, Sigma–Aldrich), triethylamine (>99%, Sigma–Aldrich), calcium hydride (CaH) (>99%, Sigma–Aldrich), chloroform (>99%, Sigma–Aldrich), and acetonitrile (MeCN) (>99%, Sigma–Aldrich) were used as received. Methyl p-toluenesulfonate (MeOTs) (>99%, Sigma–Aldrich) was distilled under dry conditions before use. Anhydrous magnesium sulfate (extra pure) and sodium hydrogen carbonate were purchased from Scharlau Chemicals. Deuterated chloroform ($CDCl_3$, 99.8%) and water (D_2O, 99.8%) were purchased from Cambridge Isotope Labs. The crosslinker *N,N'*-methylenebisacrylamide (MBA) and initiator ammonium persulfate (APS) were purchased from Sigma–Aldrich and used without further purification. Graphene oxide was synthesized from graphite powder by a modified Hummer's method.[1] The concentration of the resulting GO suspension was 1.5 mg mL^{-1} in water and was then diluted to 0.5 mg mL^{-1}.

2. Synthesis of 2,4-dimethyl-2-oxazoline macromonomers

The 2,4-dimethyl-2-oxazoline (dMeOx) monomers (enantiopure and racemic) were prepared and purified as described in the literature.[2] Each of the monomers was distilled over CaH before use. Macromonomer (MM) synthesis was achieved via well-established living cationic ring-opening polymerization (CROP). Briefly, methyl tosylate (1 mol eq 6.7 mM, 1 mL) was added to a nitrogen-purged microwave vial along with MeCN (12.36 mL, 2 M final conc.) Before the addition of the dMeOx monomer (5 eq, 33 mM, 3.3 mL), the reaction was sealed, and microwave irradiation was used to heat the solution to 14 °C for 10 mins with stirring. The reaction vessel was quickly cooled to 0 °C, and a crude sample under nitrogen flow was taken before end capping with acrylic acid (1.25 mol eq) in the presence of triethyl amine (1.4 mol eq). The polymer was dissolved in 200 mL of chloroform and washed 3x with saturated sodium hydrogen carbonate and once with brine. Anhydrous magnesium sulfate was added to the chloroform layer and was then filtered off. The chloroform was then removed under vacuum. A yellow solid gel (*S*-PdMeOx$_5$A) was obtained in 60% yield.

3. Preparation of homochiral *S*-PdMeOx/GO membrane

S-PdMeOx5A (46.2 mg) and MBA (9 mg) were dissolved in 4 mL 0.5 mg mL^{-1} GO aqueous solution, and the solution was sonicated for 10 min. After complete dissolution of the macromonomers and crosslinkers, 5 mg APS was added to the system followed by magnetic stirring. A nylon substrate (47 mm, 0.1 μm, Sterlitech Corporation) was used as the base layer of the composite membrane. To prepare the membrane, the precursor solution was filtered through a nylon substrate under a pressure of 0.098 MPa using a glass filtration apparatus and vacuum pump. The coated substrate was placed in a convection oven for free-radical polymerization at 70 °C for 24 h and then coated with the *S*-PdMeOx/GO layer via spin-coating. Specifically, the substrate was placed in a spin coater (WS-650-23B, Laurell Technologies Corporation, PA, USA), and 2 mL of precursor solution was dropped onto the membrane surface; a thin active layer was formed via spin coating at 1000 rpm for 30 s. The coated substrate was then placed in a convection oven at 70 °C for 1 h. The coating and heating were repeated three times for the fabrication of defect-free homochiral polymer-GO composite layers.

4. Adsorption experiment

The *S*-PdMeOx/GO thin film was prepared through similar procedures as the *S*-PdMeOx/GO membrane except that anodic aluminum oxide (AAO) (25 mm, 0.02 μm, Whatman™) was used as the substrate instead of nylon. A thin film was peeled off from the AAO substrate after vacuum filtration of the precursor solution, and the freestanding thin film was then coated with *S*-PdMeOx/GO active layers via spin coating. The obtained thin film was immersed in 10 mL 0.01 mol L^{-1} *R/S*-limonene solution followed by magnetic stirring for 24 h. Afterwards, the *S*-PdMeOx/GO thin film was moved and washed with ethanol to remove limonene molecules on the surface. Then, 10 mL of ethanol was used to desorb the guest molecules inside the active layers via 24 h of extraction. Finally, the concentration of limonene enantiomers in the collected solution was analyzed using gas chromatography.

5. Separation measurements

The chiral separation performance of the *S*-PdMeOx/GO membrane was measured using a diffusion apparatus. The diffusion apparatus was composed of two Teflon cells (10 mL) and a stainless-steel container for holding the cells. During the separation measurement, the membrane was placed between the two cells facing the feed side, where the active membrane area was 0.785 x 10^{-4} cm^2. The feed end was fed 10 mL 0.01 mol L^{-1} *R/S*-limonene/ethanol solution, and the permeate end was loaded with 10 mL pure ethanol. The measurement proceeded for 4 h, and 0.5 mL permeates were collected every 2 h for gas chromatography analysis.

6. Characterization

1H nuclear magnetic resonance (^{1}H NMR) spectroscopy of all samples was carried out using a Bruker AVANCE III HD 400 MHz spectrometer in deuterated solvents using an external lock and referenced internally to a resonance from residual protonated solvent. Chemical shifts (δ) are reported in parts per million (ppm). Analyses of polymer solutions were performed using a Shimadzu modular system comprising a DGU-12A degasser, an SIL-20AD automatic injector, and a 5.0 μm bead-size guard column (50 x 7.8 mm) followed by three KF-805 L columns (300 x 8 mm, bead size: 10 μm, pore size maximum: 5000 Å), an SPD-20A ultraviolet detector, and an RID-10A differential refractive index detector. A CTO-20A oven was used to maintain the columns at 40 °C. N,N-dimethylacetamide (DMAc) with 0.03% w/v LiBr was used as the eluent, and the samples were analyzed under isocratic conditions at 1 mL min^{-1}. Polystyrene standards (0.5 to 2000 kg mol^{-1}) were used for calibration. Analyte samples were filtered through 0.45 μm PTFE filters before injection. The molar mass (Mn, SEC) and dispersity (Đ) values of the samples were determined on Shimadzu LabSolutions software. Morphologies and cross-sections of the membranes were studied using a field emission scanning electron microscope (FE-SEM) (Nova NanoSEM 450, FEI, Hillsboro, OR) at a voltage of 5 kV. A Fourier transform infrared (FT-IR) spectrometer (Perkin Elmer, Spectrum Two FT-IR Spectrometer) was used to determine the chemical structure of the composite membrane. The crystalline structure of the GO-polymer membrane was analyzed using an X-ray diffraction (XRD) instrument (Bruker D8 Advance diffractometer). The composition of the membrane sample was obtained through thermogravimetric analysis (TGA) (TA SDT 650 simultaneous thermal analyzer). To prepare the sample for analysis, the polymer-GO composite layers were scraped off from the substrate. The TGA experiments were performed in a nitrogen environment at a ramping rate of 10 °C min $^{-1}$. The gas chromatography (Agilent 6850 GC series, Mulgrave, Australia) technique was used to analyze the relative concentration of enantiomers in the permeate. The GC instrument was equipped with a flame ionization detector (FID), a length of deactivated fused silica (DFS, 5 m long × 0.25 mm I.D.), and an Astec Chiraldex B-PM (Supelco, Bellefonte, PA) (30 m long × 0.12 μm film thickness × 0.25 mm I.D.) enantioselective column connected through a Siltek universal press tight connecter (Restek, Bellefonte, PA). One microliter of sample was injected for each analysis (split ratio 1:10). The initial oven temperature was set at 40 °C and then heated to 120 °C at a ramping rate of 15 °C min^{-1} and held for 20 min. The temperature was further increased to 170 °C at the same ramping rate and held for the same amount of time. The carrier gas was hydrogen, and the flow rate was 10 mL min^{-1}. Data were collected at 10 Hz, and ChemStation software (Agilent) was used for data collection and further analysis.

7. Calculation of enantioselectivity and flux

The enantioselectivity of the *S*-PdMeOx/GO composite membrane was presented using the enantiomeric excess (*ee*) value, and the *ee* value was determined by the areas of peaks of the enantiomers (A_S and A_R for the areas of the *S*- and *R*-enantiomers, respectively) from the GC responses:

$$ee\ value\ (\%) = \frac{|A_S - A_R|}{A_S + A_R} \times 100\ \%$$

The flux of *S*- and *R*-isomers as a function of time (mol m^{-2} h^{-1}) was interpreted using Fick's second law of diffusion, and the partial differential equation is stated below:

$$\frac{\partial C}{\partial t} = D\frac{\partial^2 C}{\partial x^2}$$

where C is the concentration (mol L^{-1}), t is time (s), D is the diffusion coefficient (m^2 s^{-1}), and x is the thickness of the membrane (m).
According to the boundary condition, C equals the initial concentration C_0 when x = 0, and equation (2) can be expressed in the form of

$$C(x,t) = C_0 \operatorname{erf}(\frac{x}{2\sqrt{Dt}})$$

The approximated form of the error function 'erf' is shown below:

$$erf(x) = \frac{2}{\sqrt{x}}\int_0^x e^{-t^2}dt$$

To calculate the flux of enantiomers, a plot of the concentration of each enantiomer over time was made, and through curve fitting with R^2 greater than 0.99, an exponential equation was obtained. By differentiating the exponential equation, the flux as a function of time can then be calculated for each enantiomer.

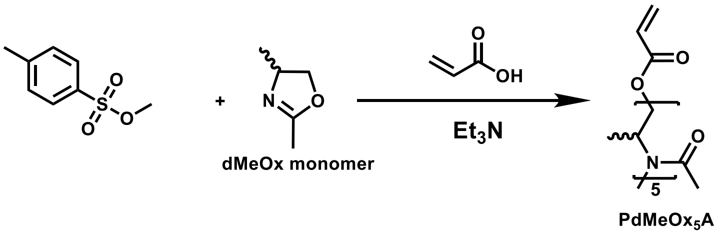

Scheme S1. Synthesis of racemic poly(2,4-dimethyl 2-oxazoline) (PdMeOx) macromonomer.

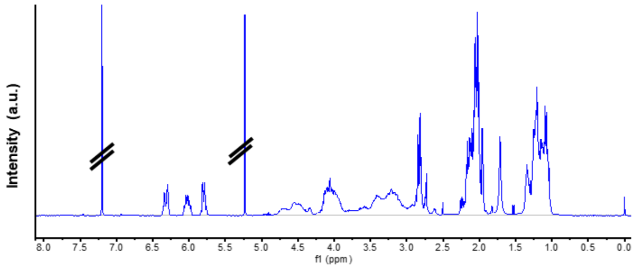

Figure S1. 1H NMR spectra (400 MHz, $CDCl_3$) of *S*-PdMeOx$_5$A.

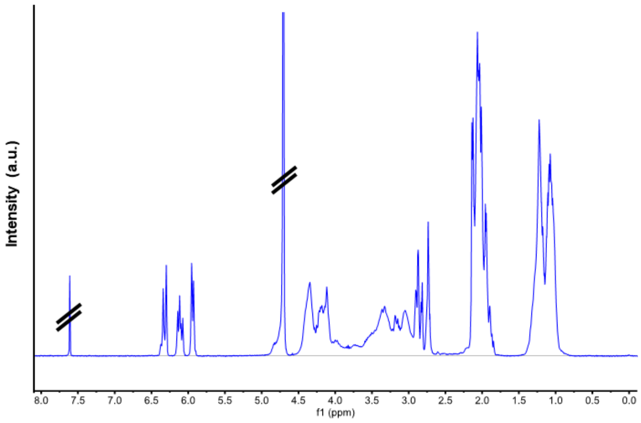

Figure S2. 1H NMR spectra (400 MHz, D_2O) of *RS*-PeMeOx$_5$A.

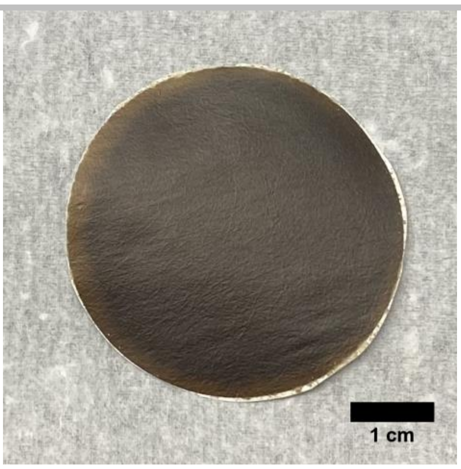

Figure S3. Digital photo of *S*-PdMeOx/GO membranes.

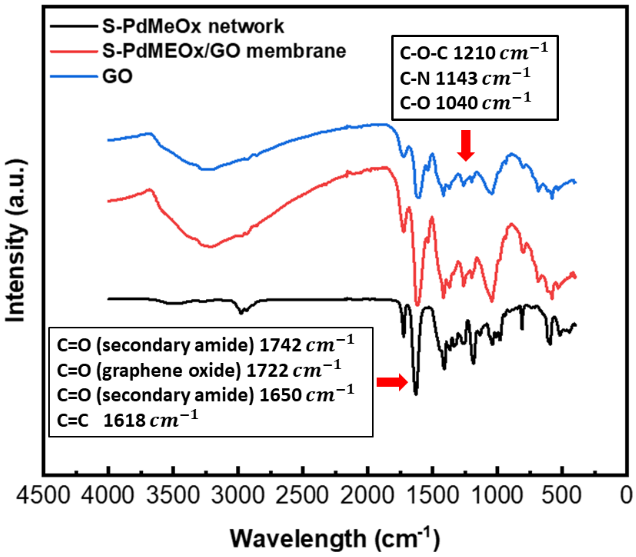

Figure S4. The Fourier transform infrared (FT-IR) spectra of GO laminates (blue), the *S*-PdMeOx polymer network (black), and *S*-PdMeOx/GO membrane (red).

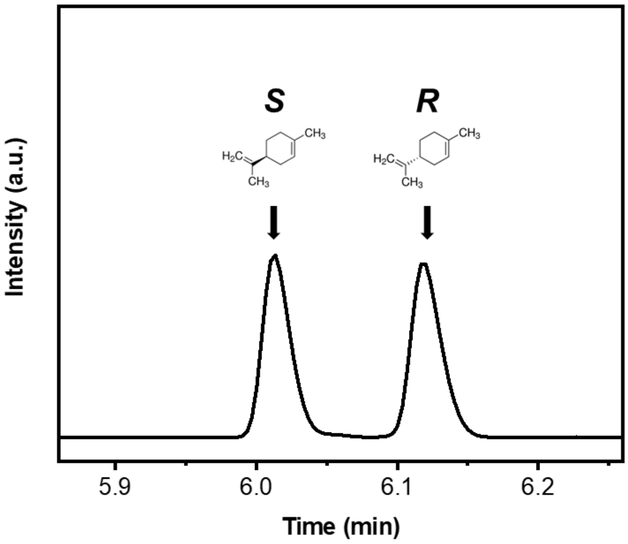

Figure S5. Gas chromatograms of 0.01 mol L^{-1} *R/S*-limonene/ethanol standard solution.

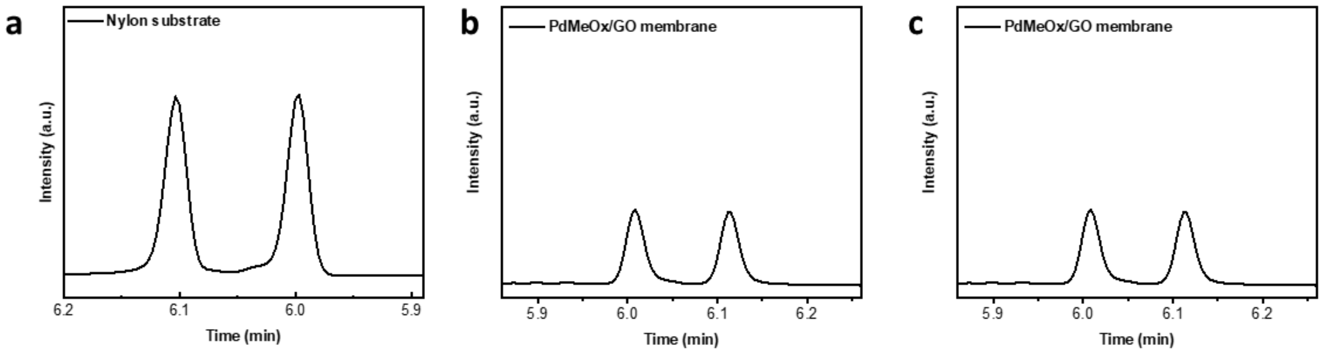

Figure S6. Gas chromatograms of resolved limonene molecules after 2 h using a) pure nylon substrate, b) GO nanosheets (supported by nylon substrate), and c) PdMeOx/GO membrane under the condition of 0.01 mol L^{-1} *R/S*-limonene/ethanol as feed solution at room temperature.

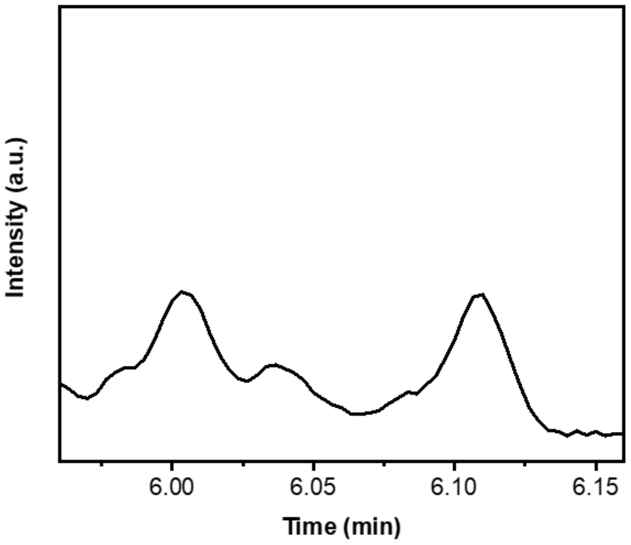

Figure S7. Gas chromatograms of *R/S*-limonene adsorbed by the *S*-PdMeOx/GO active layer (24 h).

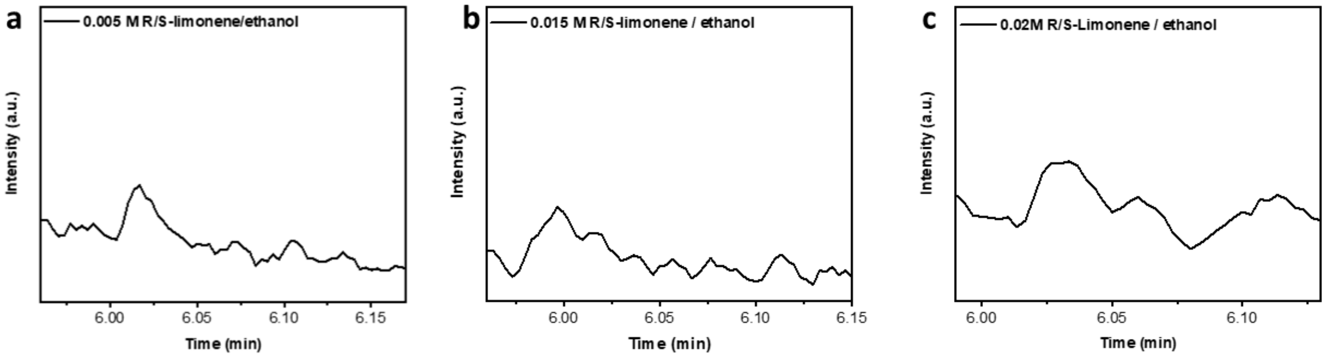

Figure S8. Gas chromatograms of resolved limonene enantiomers after 2 h using the *S*-PdMeOx/GO membrane under the conditions of (a) 0.005 mol L^{-1}, (b) 0.015 mol L^{-1}, and c) 0.02 mol L^{-1} *R/S*-limonene/ethanol as feed solution at room temperature.

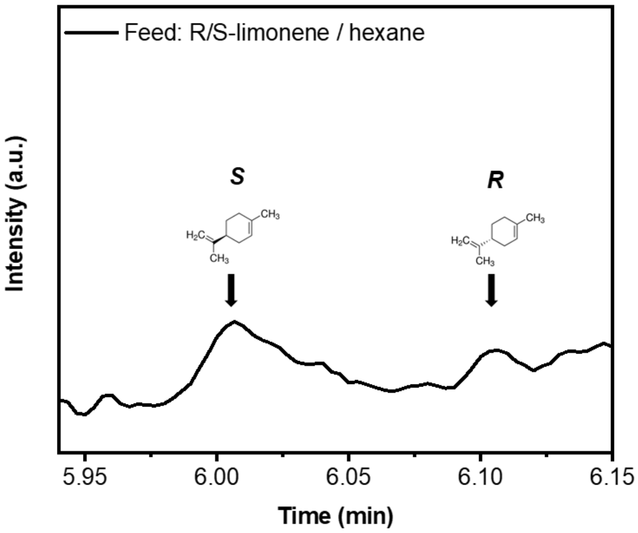

Figure S9. Gas chromatograms of resolved limonene enantiomers after 4 h using the *S*-PdMeOx/GO membrane under the conditions of 0.01 mol L^{-1} *R/S*-limonene/hexane as the feed solution at room temperature.

Table S1. Macromonomer molar masses and dispersity as measured by SEC.

Macromonomer	M_n (SEC)[a] (g mol^{-1})	Dispersity[b] (Đ)
***S*-PdMeOx$_5$A**	830	1.13
***RS*-PdMeOx$_5$A**	760	1.24

[a] Number molar mass average (M_n) as obtained from SEC (DMAc, RI detection, and PS calibration).
[b] Dispersity as calculated from the weight molar mass average M_w divided by M_n (obtained from SEC).

Table S2. Enantioselectivity and solute flux of the *S*-PdMeOx/GO membrane with permeation time.

Feed Concentration (mol L^{-1})	Permeation Time (h)	Enantioselectivity (% *ee*)	Flux (*S*-(-)-limonene) (mmol m^{-2} h^{-1})	Flux (*R*-(+)-limonene) (mmol m^{-2} h^{-1})	Standard Error of *ee* value (%)
**0.01**	2	98.3	0.32	0.001	1.7
**0.01**	4	98.5	0.24	0.0002	1.5
**0.005**	2	100	0.08	0	-[a]
**0.015**	2	65.3	0.49	0.1	3.4
**0.02**	2	50	0.88	0.29	-

[a] Indicate relevant data not available.

Table S3. Comparison of the chiral separation performance of the *S*-PdMeOx/GO membrane with reported representative enantioselective membranes.

Membrane	Feed racemic mixture	Driving force for separation	Max. *ee* value of permeate solution	Flux (mmol m^{-2} h^{-1})	Ref.
***S*-PdMeOx/GO**	0.01 mol L^{-1} *R/S*-limonene ethanol solution	Concentration gradient	98.3±1.7%	0.32	This work
	0.01 mol L^{-1} *R/S*-limonene hexane solution	Concentration gradient	54.6%	0.31	
**Cellulose acetate (CA)/cellulose acetate propionate (CAP)**	*Trans*-stilbene oxide racemate	Pressure driven	97%	0.09	[3]
**Cellulose membrane**	Racemic mandelic acid	Pressure driven	93%	0.86	[4]
**β-cyclodextrin glutaraldehyde crosslinked polysulfone membrane (β-CDXM)**	Racemic phenylalanine (Phe)	Reverse osmosis testing module	81.1%	60	[5]
	Racemic tryptophan (Trp)		49.2%	39.2	
**β-cyclodextrin-silica nanochannel membrane**	Racemic tryptophan	Concentration gradient	71.3%	-[a]	[6]
**(+)-PIM-CN membrane**	TTSBI	Concentration gradient	87%	4.8x10^{-3}	[7]
	(*R,S*)-BINOL	Concentration gradient	53%	0.6x10^{-4}	
	(*R,S*)-Mandelic acid	Concentration gradient	32%	4.6x10^{-4}	
***S*- and *R*-FTPI membrane**	1,1'-binaphthyl-2,2'-diol (rac-Binol)	Concentration gradient	96.3% (*R*-FTPI membrane)	2.44	[8]
	2-naphthyl-1-ethanol (rac-NpOH)	Concentration gradient	11.1% (*R*-FTPI membrane)	69.7	
	Mandelic acid (rac-Man)	Concentration gradient	7.4% (*R*-FTPI membrane)	26.9	
**CDCMP-1**	0.0012 mol L^{-1} Racemic	Concentration gradient	94.1%	0.8x10^{-3}	[9]

	phenylalanine aqueous solution				
**C_DCMP-2**	0.003 mol L^{-1} Racemic phenylalanine aqueous solution	Concentration gradient	85.5%	$1.29x10^{-5}$	
**C_LCMP-1**	0.003 mol L^{-1} Racemic phenylalanine aqueous solution	Concentration gradient	64.6%	$2.98x10^{-5}$	
**GCN-CSA membrane**	0.06 mol L^{-1} *R*/*S*-limonene ethanol solution	Concentration gradient	89%	-[a]	[10]
**Glu-GO membrane**	$0.5x10^{-4}$ mol L^{-1} 3,4-Dihydroxy-phenylalanine aqueous solution	Pressure driven	34.6%	0.21	[11]
**L-His-ZIF-8 membrane**	0.008 mol L^{-1} 1-phenylethanol ethanol solution	Concentration gradient	76.0%	5.1	[12]

[a] Indicates relevant data not available

References

[1] a) D. A. Dikin, S. Stankovich, E. J. Zimney, R. D. Piner, G. H. Dommett, G. Evmenenko, S. T. Nguyen, R. S. Ruoff, *Nature* **2007**, *448*, 457-460; b) W. S. H. Jr, R. E. Offeman, *J. Am. Chem. Soc.* **1958**, *80*, 1339.
[2] R. Luxenhofer, S. Huber, J. Hytry, J. Tong, A. V. Kabanov, R. Jordan, *J. Polym. Sci., Part A: Polym. Chem.* **2013**, *51*, 732-738.
[3] L. Z. Flores-López, J. Caloca, E. Rogel-Hernández, H. Espinoza-Gomez, *Cellulose* **2014**, *21*, 1987-1995.
[4] C. Ma, X. L. Xu, P. Ai, S. M. Xie, Y. C. Lv, H. Q. Shan, L. M. Yuan, *Chirality* **2011**, *23*, 379-382.
[5] K. Singh, P. G. Ingole, H. C. Bajaj, H. Gupta, *Desalination* **2012**, *298*, 13-21.
[6] Y. Liu, P. Li, L. Xie, D. Fan, S. Huang, *J. Membr. Sci.* **2014**, *453*, 12-17.
[7] X. Weng, J. E. Baez, M. Khiterer, M. Y. Hoe, Z. Bao, K. J. Shea, *Angew. Chem.* **2015**, *54*, 11214-11218.
[8] Q. P. Zhang, Z. Wang, Z. W. Zhang, T. L. Zhai, J. J. Chen, H. Ma, B. Tan, C. Zhang, *Angew. Chem.* **2021**, *60*, 12781-12785.
[9] Y. Huang, Y. Zang, L. Xu, T. Lei, J. Cui, Y. Xie, J. Wang, H. Jia, F. Miao, *Sep. Purif. Technol.* **2021**, *266*, 118529-118539.

[10] Y. Wang, N. Wu, Y. Wang, H. Ma, J. Zhang, L. Xu, M. K. Albolkany, B. Liu, *Nat. Commun.* **2019**, *10*, 2500.
[11] C. Meng, Y. Sheng, Q. Chen, H. Tan, H. Liu, *J. Membr. Sci.* **2017**, *526*, 25-31.
[12] J. Y. Chan, H. Zhang, Y. Nolvachai, Y. Hu, H. Zhu, M. Forsyth, Q. Gu, D. E. Hoke, X. Zhang, P. J. Marriot, H. Wang, *Angew. Chem.* **2018**, *57*, 17130-17134.
